# The effect of quitting water pipe during pregnancy on anthropometric measurements at birth: a population-based prospective cohort study in the south of Iran

**DOI:** 10.1186/s12884-020-02948-2

**Published:** 2020-04-22

**Authors:** Shahrzad Nematollahi, Koroush Holakouie-Naieni, Abdolhossain Madani, Hossein Shabkhiz, Elham Torabi, Samane Lotfi

**Affiliations:** 1 Men’s Health and Reproductive Health Research center, Shahid Beheshti University of Medical Sciences, Tehran, Iran; 2 Department of Epidemiology & Biostatistics, School of Public Health, Tehran University of Medical Science, Tehran, Iran; 3 School of Public Health, Hormozgan University of Medical Sciences, Bandar Abbas, Iran; 4 Bandar Abbas Heath Education & Research Station, School of Public Health, Tehran University of Medical Science, Tehran, Iran; 5 Department of Epidemiology and Public Health, Iran University of Medical Sciences, Tehran, Iran

**Keywords:** Water pipe smoking, Anthropometry, Prospective cohort study, Bandar Abbas

## Abstract

**Background:**

Evidence regarding health effects of tobacco cessation during pregnancy is mainly restricted to cigarette while water pipe is the preferred method of tobacco smoking among women in the Middle-East. The present study aimed to assess the effects of cessation of water pipe during pregnancy on birth anthropometric measures in the south of Iran.

**Methods:**

Data on 1120 singleton pregnancies (response rate = 93.4%) from a population-based prospective cohort study in suburban communities in Bandar Abbas city was used. Based on water pipe smoking status, the study subjects were categorized into: 1) those who never smoke water pipe (never smoker); 2) those who stopped water pipe during pregnancy and resumed it postpartum (quitters); 3) those who continued smoking water pipe during their pregnancy (always smokers). The Generalized Linear Models (GLMs) were utilized for the analyses.

**Results:**

Compared to never smokers, quitting water pipe in pregnancy decreased mean birthweight of infants by 99.30 g (β:-99.30, 95%CI:-204.35,-5.75) and an additional decrease of 37.83 g occurred in infants of always smokers (β:-137.13;95%CI:− 262.21,-12.05). Means of birth length did not significantly differ among the three water pipe groups. Means of head circumference, however, significantly increased by 0.79 cm in infants of always smokers (β:079,95%CI:0.13,1.45).

**Conclusion:**

Quitting water pipe during pregnancy had positive effects on infant growth, especially birth weight. Awareness campaigns about health benefits of quitting water pipe during routine prenatal checkups and integration of active follow-up visits into prenatal care protocols for smoking mothers are provided.

## Background

Due to the well-established adverse effects of cigarette smoking on pregnancy outcomes [1], attention now has been shifted to other types of tobacco use, such as water-pipe smoking. The tradition of water pipe (also known as narghile, hookah, and shisha) dates back 400 years in Turkey, India, and Iran [2] and it is widely believed to be a less harmful form of tobacco consumption and a safer alternative to cigarette smoking [3]. In the south of Iran, reports have shown that approximately 8.0–14.0% of pregnant women use water pipe regularly [4, 5]. Adverse effects of water pipe on fetal growth have been the focus of very limited research in Iran. Cross-sectional research shows that compared to non-smokers, the odds of intrauterine growth retardation would increase by 2–3.5 times in water pipe smokers [6, 7]. On the other hand, a negative correlation is reported between infant’s mean birth weight and extent of maternal cigarette smoking [8]. The promising health outcomes of cessation of smoking during gestational period have been reported in a few studies, mostly conducted in developed countries [9, 10]. At the same time, water pipe has been gaining popularity among Iranian women, which necessitates investigation on its health effects with special consideration to reproductive outcomes. To the best of our knowledge; however, there is no epidemiologic evidence on the effects of water pipe cessation on fetal growth among Iranian population. Such epidemiologic evidence is required in order to make recommendations to quit smoking during pregnancy period and to be integrated into routine prenatal healthcare services. Therefore, the aim of the present study was to estimate the effects of water pipe cessation during pregnancy on birth anthropometric measures in the south of Iran.

## Methods

**Fig. 1:**
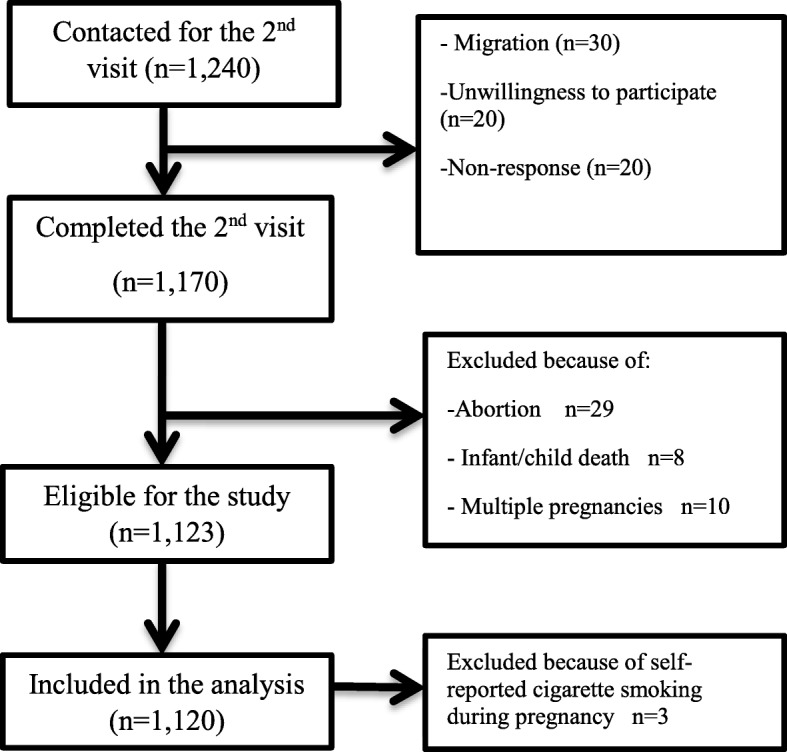
Flowchart of the BAPC subjects participated in the present study

We used data of a prospective cohort study entitled “A population-based prospective cohort study to identify contributors of mother and child health in suburban communities” in Bandar Abbas city, which is abbreviated as Bandar Abbas Pregnancy Cohort (BAPC). BAPC aims to quantify the effects of lifestyle and environmental factors on maternal and child health among residents of suburban communities in Bandar Abbas, an unindustrialized region in the south of Iran. The study subjects were recruited through home-by-home sampling scheme. All pregnant women above the age of 16 who were residing in the study area for at least 6 months prior to the interview were eligible to participate. Exclusion criteria was defined as pregnant women who had medically-assisted conception or were unwilling to participate. Follow-up visits were performed at home by two trained interviewers. The study protocol was ethically approved and financially funded by the National Institute for Medical Research Development (with the approval code: 943607 and ethical code: N. IR.NIMAD.REC.1396.205). The details of the methodology have been published elsewhere [11]. The present study used data on 1120 successful singleton pregnancies (response rate = 93.45%) (Fig. 1). Explanatory variables were maternal demographics, household monthly expenditure and obstetrics history. According to the national guideline, the pattern of receiving prenatal healthcare services was categorized into regular (at least 9 visits to healthcare centers), irregular (less than 9 visits) and not receiving. The self-rated health was measured using the following question:“ How would you rate your general health status?” on a 5-point scale ranging from ‘very good’ (a score of 5) to ‘very bad’ (a score of 1) [12]. The Socio-Economic Status (SES) was calculated by the Principal Component Analysis (PCA) method on ownership of nine household assets. The study outcomes were newborn’s birth anthropometric measures including weight (in gram), length (in centimeter) and head circumference (in centimeter) and were collected from infant’s vaccination card. The exposure of interest was the pattern of water-pipe smoking during the current pregnancy, which was measured by a checklist during the first visit in pregnancy. To detect changes in water pipe smoking, an additional question during the postpartum visit asked the subjects:“ *Did you stop smoking water pipe because you realized you were pregnant?*”. The pattern of water pipe smoking was categorized into three distinct groups: 1) those who never smoke water pipe (never smoker); 2) those who stopped water pipe during pregnancy and resumed their smoking behavior postpartum (quitters); 3) those who continued water pipe regardless of their pregnancy (always smokers). Second-hand water pipe smoking was also categorized into “yes/no”, which was applied for both pregnancy and postpartum period. Bivariate comparisons were performed using Chi-square and independent samples t-test. Confounder selection was based on the change-in-estimate strategy. Accordingly, covariates for which change in exposure effect estimate (i.e. regression coefficient) fell outside the range of 10% were considered as the potential confounders [13]. The Generalized Linear Models (GLMs) with Gaussian family and Identity link function and robust standard errors were utilized to measure the effects of pattern of water pipe smoking on the study outcomes. All the analyses were performed using STATA version 14 (Stata Corp., College Station, TX, USA). For the final model, *p*-values< 0.05 were considered as statistically significant.

## Results

Of 1120 subjects, more than half (*n* = 583, 52%) were 26–35 years old and 81% (*n* = 916) had education level below high-school diploma. Almost all of the subjects belonged to families with four or less members (*n* = 1005, 89.7%). More than half (*n* = 628, 56%) of the subjects reported ownership of less than 4 assets, and 57% (*n* = 639) reported a monthly expenditure of less than 41US$. Overall, 969 subjects (86.5%) never smoked water pipe during their pregnancy. Sixty-two subjects (5.5%), which was equivalent to 58% of the smokers, used water pipe both in pregnancy and postpartum period and 89 subjects (7.9%), which was equivalent to 42% of smokers, quitted water pipe because of pregnancy and resumed it in postpartum. Among the always smokers, the mean (SD) age at smoking initiation was 18.4 (4.2) years and 62% of the smokers (*n* = 40) had one water pipe session per day. Compared to boy infants, mean birth weight of girl infants was significantly lower (mean = 3100.7 ± 540 vs. 2943.6 ± 506 g; *p* < 0.001). The mean birthweight was also significantly higher in term infants in comparison to preterm infants (mean = 3067.3 ± 465 vs. 2233.3 ± 780 g; *p* < 0.001). More specifically, median and inter-quartile range of birth weight according to the gestational age at delivery was 3000 and 610 g for term infants and 2220 and 1000 g for preterm infants, respectively. Mean birth weight (SD) of infants of women with excellent self-rated health was 3022 (523) grams, which was significantly higher in comparison to mothers with poor or average self-rated health (2978 (603) grams in average self-rated health and 3400 (507) grams in poor self-rated health, *p* = 0.060). On average, infants from families with more than four members had significantly shorter birth length compared to infants from families with less than four members (48.1 ± 2.74 vs. 48.6 ± 2.52; *p* = 0.0006). Furthermore, compared to families with less than 4 members, mean head circumference was significantly lower in infants from families with more than 4 members (41.07 ± 2.5 vs. 41.4 ± 2.6, *p* = 0.007). The mean birth length of infants born by Cesarean section delivery was significantly lower than those who were born by vaginal delivery (48.2 ± 2.8 vs. 48.5 ± 2.4, *p* = 0.010). Newborns of never smokers had the highest mean (SD) birth weight (3035 ± 527 g) in comparison to newborns of quitters and always smokers (2914.1 ± 510 and 2848.3 ± 574 g, respectively, *p* = 0.0046). The mean (SD) birth length was 48.4(2.6) centimeters in newborns of never smokers, 47.9 (2.5) centimeters in newborns of quitters and 48(2.9) centimeters in newborns of always smokers. Categorization on the time of smoking cessation among quitters showed that the mean (SD) birthweight was 2901.81(102), 2951.29(102), and 2894.94(82) grams in infants of mothers who quitted water pipe in the first, second, and third gestational trimester, respectively. The difference of mean birth weight according to the time of smoking cessation was not statistically significant (*p* = 0.903).

The mean (SD) head circumference was 41.2 (2.5) centimeters in newborns of never smokers, 41.3(2.6) centimeters in newborns of quitters and 41.9(2.5) centimeters in newborns of always smokers. Means of length and head circumference were not statistically significantly different among the three categories of water pipe smoking (*p* = 0.091 and 0.1007, respectively) (Table 1). The results of the final generalized linear model for birth weight showed that compared to never smokers, quitting water pipe due to pregnancy decreased mean birthweight by 99.30 g (β: -99.30, 95%CI: − 204.35, − 5.75) while continuing water pipe smoking during pregnancy additionally decreased mean birthweight by 37.83 g (β: -137.13; 95%CI: − 262.21, − 12.05). The final model for mean birth length did not yield statistically significant results. The final model for mean head circumference showed that compared to non-smokers, mean head circumference increased by 0.79 cm in infants of always smoking mothers (β:079, 95%CI: 0.13, 1.45) (Table 2).

**Table 1:** Newborn’s anthropometric measures at birth by maternal characteristics in the BAPC project

Variable	N(%)	Weight in grams Mean (SD)	Length in centimeters Mean (SD)	Head circumference in centimeters Mean (SD)
**Demographic characteristics**				
**Maternal age**				
> 15	3 (0.27)	2666.66 (351.18)	47.33 (2.08)	40.97 (0.19)
16–25	439 (39.20)	3033.03 (499.29)	48.50 (2.48)	41.31 (2.67)
26–35	583 (52.05)	3015.00 (551.12)	48.30 (2.84)	41.26 (2.48)
< 35	95 (8.48)	3040.42 (537.74)	48.71 (1.95)	41.36 (2.70)
**Maternal education**				
illiterate	51 (4.55)	3121.96 (704.28)	48.29 (2.80)	41.37 (2.70)
Below diploma	916 (81.79)	3016.72 (531.46)	48.38 (2.61)	41.29 (2.59)
academic	153 (13.66)	3029.73 (444.95)	48.66 (2.72)	41.23 (2.40)
**Maternal job**				
With income	28 (2.5)	2987.14 (405.61)	48.53 (2.45)	40.86 (2.90)
Without income	1092 (97.50)	3024.22 (532.53)	48.41 (2.64)	41.30 (2.56)
**SES (Item)**				
> 4	628 (56.07)	3028.14 (538.14)	48.64 (2.52)	41.46 (2.60)
≤ 4	492 (43.93)	3017.10 (519.03)	48.12 (2.74)	41.07 (2.52)
**Household Monthly expenditure** (US$)				
≤ 41.00	639 (57.05)	3026.04 (522.58)	48.24 (2.63)	41.30 (2.61)
41.00–83.99	220 (19.64)	2992.59 (514.07)	48.64 (2.50)	41.48 (2.51)
84.00–166.99	215 (19.20)	3044.81 (577.72)	48.63 (2.73)	41.07 (2.47)
> 167.00	46 (4.11)	3031.30 (472.18)	48.79 (2.69)	41.18 (2.73)
**Family size**				
≤ 4	1005 (89.73)	3017.02 (537.95)	48.60 (2.52)	41.40 (2.58)
> 4	115 (10.27)	3078.08 (448.68)	48.10 (2.74)*	41.07 (2.53)*
**Pregnancy characteristics**				
**Receiving prenatal care from Healthcare centers**				
Regular	391 (34.91)	3028.63 (524.22)	48.47 (2.36)	41.19 (2.53)
Irregular	717 (64)	3018.02 (534.38)	48.37 (2.79)	41.34 (2.60)
Not received	12 (1.07)	3164.16 (423.24)	48.70 (1.25)	41.09 (1.77)
**Maternal Self-rated health**				
Poor/very poor	10 (0.89)	3400.00 (507.71)	47.95 (3.93)	40.52 (2.80)
Medium	77 (6.88)	2978.83 (603.31)	48.27 (2.67)	41.26 (2.43)
good /excellent	1033 (92.23)	3022.96 (523.04)*	48.43 (2.62)	41.30 (2.58)
**Anemia during pregnancy**				
Yes	311 (27.77)	3020.87 (487.68)	48.46 (2.37)	41.29 (2.46)
No	809 (72.23)	3024.22 (545.17)	48.39 (2.73)	41.29 (2.61)
**Delivery type**				
Vaginal	647 (57.77)	3038.44 (472.93)	48.57 (2.48)	41.34 (2.61)
Cesarean	473 (42.23)	3002.56 (598.45)	48.20 (2.82)*	41.22 (2.52)
**Infant sex**				
Boy	568 (50.71)	3100.70 (540.63)	48.63 (2.76)	41.47 (2.68)
Girl	552 (49.29)	2943.64 (506.32)*	48.18 (2.48)	41.10 (2.44)
**Preterm delivery**				
Yes	70 (6.25)	2233.35 (780.19)	45.37 (4.81)	41.12 (3.26)
No	1050 (93.75)	3067.34 (465.62)*	48.62 (2.28)	41.30 (2.52)
**Water pipe smoking**				
Never smoker	969 (86.52)	3035.18 (527.08)	48.48 (2.62)	41.24 (2.57)
Quitter	89 (7.95)	2914.12 (510.89)	47.96 (2.51)	41.35 (2.64)
Always Smoker	62 (5.54)	2848.30 (574.78)	48.00 (2.90)	41.96 (2.50)
**Second-hand tobacco smoking**				
Yes	183 (16.40)	3042.30 (502.80)	48.30 (2.85)	41.27 (2.35)
No	937 (83.60)	3009.90 (535.90)	48.40 (2.59)	41.29 (2.61)

**Table 2:** Adjusted effects of quitting water pipe smoking on infant anthropometric measures

Water pipe pattern	Birthweight ^**a**^			Birth length ^**b**^			Birth head circumference^**c**^		
	β	95%CI	*p*	β	95%CI	*p*		95%CI	*p*
Non- smoker	Reference			Reference			Reference		
Always smoker	-137.13	-262.21,-12.05	0.032	−0.23	−0.87, 0.41	0.481	0.79	0.13, 1.45	0.018
Quitter	−99.30	−204.35, −5.75	0.064	−0.42	−0.96, 0.11	0.122	0.16	−0.39, 0.71	0.571

^a^ adjusted for: self-rated health, infant sex, family size, SES, preterm delivery, secondhand water pipe smoking

^b^ adjusted for: infant sex, SES, delivery type, secondhand water pipe smoking

^c^ adjusted for: infant sex, SES, preterm delivery, secondhand water pipe smoking

## Discussion

Maternal tobacco smoking during pregnancy is a well-recognized cause of fetal growth restriction and preterm birth [14]. Tobacco smoking during pregnancy can increase the risk of fetal growth restrictions up to two times; where nearly one-quarter of all infants with growth restriction can be attributed to tobacco smoking [15, 16]. Regardless of the underlying theory for the effects of smoking cessation on infant health, all the previous studies focused on cigarette as the mode of tobacco smoking. Our study attempted to provide a clearer picture on the effects of water pipe cessation on pregnancy outcomes. In spite of similar health consequences of various types of tobacco smoking, water pipe has unique health effects, which could not be fully captured by studies on cigarette smoking [17]. In our large population-based cohort study, we found that 5.5% of the subjects were always smokers and nearly 8% of the subjects quitted water pipe following pregnancy notification. Our study showed that continuation of water pipe smoking during pregnancy was associated with a substantial reduction in birth weight of the infant. We found that 42% of water pipe smokers (8% of all study subjects) quit water pipe smoking following pregnancy occurrence. Our study also showed that the mean birth weight of infants of quitters and always smokers was 99 and 137 g lower than mean birth weight of infants of never smokers, respectively. The observed results were biologically plausible. The effects of maternal tobacco smoking on fetal growth are complex and mainly grounded on two theoretical mechanisms. It may result from an interaction between genetic traits and direct toxic effects of nicotine, or may be due to the placental-smoking interaction. Positive effects of cessation of cigarette smoking on pregnancy outcomes have been reported in a few studies [10, 18]. The results of these investigations; however, relied heavily on the selected theoretical mechanism. In other words, early cessation of tobacco smoking would result in diluted effect in the former mechanism; whereas a critical window of exposure emerged in the first trimester in the latter one [18, 19]. Setting aside different concepts, both aforementioned theories imply that the time of cessation might be an important predictor in the pathway of smoking cessation on pregnancy outcomes. Our findings supported the premise that smoking impedes growth and fetal weight gain and reinforced prior conclusions that quitting tobacco smoking will bring about major improvement in fetal growth and birth anthropometries. Consistently, *Vilalbi* et.al reported lower birth weight among infants of mothers who continued cigarette smoking during pregnancy in Spain. The authors concluded that the effects of smoking was most prominent for intra-uterine growth retardation [20]. The results of a population-based prospective cohort study in the Netherlands (the Generation R Study) showed that although pre-conception active cigarette smoking was not associated with adverse pregnancy outcomes; continued active smoking in pregnancy increased the odds of low birthweight by 75%. The authors also reported that quitting smoking in early pregnancy was associated with a higher birthweight compared to smoking continuation [21].

Notwithstanding, the present study has provided a novel piece of information indicating that quitting water pipe smoking during pregnancy might substantially improve fetal growth in water pipe smokers. The estimated effect of quitting water pipe on birth anthropometries remained even after adjusting on preterm birth and second-hand water-pipe smoking, implying the extent to which primary prevention on smoking cessation may be beneficial for all pregnant women. More specifically, our data showed that only 35% of the study subjects received regular prenatal checkups from healthcare centers, defined as nine routine checkups throughout pregnancy. In other words, more than two-thirds of pregnant women in our sample received prenatal care from private physicians or did not receive it at all. Interestingly, the proportion of women not receiving prenatal care from any sources was significantly higher among always smokers; whereas, never smokers received the care more frequently from healthcare centers (data not shown). This observation has an important implication. Encouragement to initiate prenatal healthcare services as soon as possible may assist pregnant women in making the decision to quit water pipe. Such awareness programs should be well-targeted to pregnant women from the lowest socio-economic households. To elaborate more, our previous work showed that women with lower socio-economic status had lower knowledge regarding health effects of water pipe and higher unfavorable attitude towards water pipe smoking [3]. In case of cigarette smoking, it is shown that to achieve the same risk of growth restriction as non-smoking mothers, quitting must be adopted before conception [9, 10], although it is still efficient at earlier points during pregnancy [18]. Having a similar logic for water pipe smoking, cessation programs should focus on benefits of quitting in preconception period or as early in pregnancy as possible.

Our study was among the first attempts to quantify birth anthropometric measures following various patterns of water pipe smoking during pregnancy. Our study used data from a population-based prospective cohort study, which guaranteed suitable external generalizability to a wider population of pregnant women in the south of Iran. Large sample size (*n* = 1120) provided satisfactory statistical power to strengthen the precision of our estimates. While previous studies mostly evaluated a single birth outcome such as birthweight, we studied all the three standard anthropometric measures, including weight, length, and head circumference.

### Limitations

In the absence of a ubiquitous valid tool, we used a self-report checklist to measure water pipe smoking during pregnancy. Therefore, observed differences in the estimated prevalence of water pipe smokers in our study with other estimates may be due to measurement biases, mainly reporting bias, stemmed from use of different self-report tools. Moreover, pregnant women who had medically assisted conception were excluded from the BAPC project. Hence, the estimated effects of water pipe on birth anthropometries should be interpreted with caution for this subgroup of women.

## Conclusions

Quitting water pipe during pregnancy had positive effects on birth anthropometric measures, especially birth weight. Some suggestions are provided including integration of information regarding health benefits of quitting smoking into routine prenatal healthcare services and development of awareness programs to encourage smokers to quit early in pregnancy.

## Data Availability

The dataset used and/or analyzed during the current study is available from the corresponding author on reasonable request.

## Abbreviations

GLMs: Generalized Linear Models
BAPC: Bandar Abbas Pregnancy Cohort
SES: Socio-Economic Status
SD: Standard Deviation
95%CI: 95% Confidence Intervals
